# Role of Impurity Sulphur in the Ductility Trough of Austenitic Iron–Nickel Alloys

**DOI:** 10.3390/ma13030539

**Published:** 2020-01-23

**Authors:** Frédéric Christien

**Affiliations:** 1 Mines Saint-Etienne, Univ Lyon, CNRS, UMR 5307 LGF, Centre SMS, F - 42023 Saint-Etienne, France; frederic.christien@emse.fr; Tel.: +33-477-420-018; 2 Institut des Matériaux Jean Rouxel (IMN), Université de Nantes, Polytech Nantes, BP 50609, CEDEX 3, 44306 Nantes, France

**Keywords:** ductility trough, hot-shortness, intermediate temperature embrittlement, ductility dip cracking, sulphur, nickel alloys

## Abstract

The role of impurity sulphur in the ductility trough of iron–nickel (Fe–Ni) alloys is investigated using hot tensile tests. A strong detrimental effect of some ppm levels of sulphur is demonstrated. In addition, it is shown that, in the ductility trough, material failure occurs through subcritical grain boundary crack propagation, involving dynamic embrittlement at the crack tip, due to the sulphur. Very high intergranular crack growth rates are observed. This is possible because plastic deformation accelerates the transport of sulphur to the crack tip, by several orders of magnitude, compared to normal bulk diffusion. The ductility is recovered at high strain rates, which correlates with a decrease in the sulphur concentration measured on the fracture surface. It is suggested that the main mechanism of sulphur transport is dragging by moving dislocations.

## 1. Introduction

Austenitic materials usually have a good ductility, which makes it possible to cold- or hot-deform them. In some circumstances, however, this ductility can be reduced at temperatures typically between 0.5 and 0.8 *T_M_* (melting temperature). This phenomenon is referred to as a “ductility trough”, “loss of ductility”, “hot-shortness”, “intermediate temperature embrittlement” or, sometimes, “ductility dip cracking (DDC)”, the latter being more popular in the field of welding. One very specific feature of ductility trough is that it is always associated with the occurrence of a grain boundary fracture. Many studies have shown the prime importance of trace elements, especially impurity sulphur, in the ductility trough of steels [1,2], nickel alloys [3,4,5] and copper alloys (see, for example, the review paper by Laporte and Mortensen [6]). Some of those early works on ductility troughs were conducted on model alloys of a very well-controlled purity at the ppm level, so as to identify the effects of individual elements. However, more recently, other microstructural features, like intergranular precipitates, were stated to have the most significant effect on the ductility trough of some nickel alloys [7,8]. It was even argued that, for some nickel alloys, no effect from impurity sulphur was expected in the ductility trough [7,8]. Some researchers have recently questioned the role of sulphur in the ductility trough—first, because of the absence of a clear correlation between the nominal sulphur content of industrial alloys and the intensity of their ductility troughs [7,8] and, second, because of the extreme difficulty, or even the impossibility, to demonstrate the presence of such impurities segregated at the grain boundaries [9]. It was then sometimes concluded that, as no grain boundary segregation was observed, the impurity could not be the cause of the ductility trough.

It must be noted that most of the recent studies (for example, [7,8,9]) implicitly assume that the material failure in the ductility trough is obtained from a critical fracture, i.e., with a crack propagating at a very high speed, preventing any dynamic phenomenon at the crack tip. However, another scenario for the ductility trough is possible, involving subcritical crack growth, with dynamic interactions at the propagating crack tip. In this subcritical mechanism, the impurity can be active at the crack tip, but is not necessarily segregated at the grain boundaries in the whole microstructure. This scenario is inspired from the grain boundary fracture observed in metallic materials in the case of environmentally assisted degradation, like stress corrosion cracking, hydrogen embrittlement or hot corrosion, where solutes can be dynamically active at the crack tip and thus promote its subcritical propagation.

Austenitic iron–nickel alloys have outstanding elastic and magnetic properties. They are used in a variety of applications for their tuneable thermal expansion coefficient, Curie temperature and thermoelastic coefficient, as well as their high magnetic permeability or their magnetostrictive ability [10]. Their nickel content can vary from about 30 wt.% to more than 70 wt.%, depending on the targeted property.

In this work, the ductility trough of austenitic Fe–Ni 42 alloys with different sulphur contents were studied using hot tensile testing. The results are discussed in the frame of a dynamic type of embrittlement, where the sulphur is active at the crack tip.

## 2. Materials and Methods

Two materials with different sulphur contents were used. They were fabricated using vacuum induction melting, followed by casting into 80 kg ingots. Their composition is given in Table 1. The raw materials used for the fabrication were high-purity iron (Fe), nickel (Ni), manganese (Mn) and silicon (Si). The sulphur (S) content was obtained using controlled additions of iron sulphide (FeS_2_) to the melt. The carbon (C) content indicated in Table 1 is due to impurity carbon in the raw materials used. After fabrication, the targeted composition was checked using X-ray fluorescence for Fe, Ni, Mn and Si and glow discharge mass spectrometry (GDMS) for S and C. The two alloys were provided in the as-solidified state. They have a similar millimetric grain size. The tensile specimens were cut from the equiaxed part of the ingot. Tensile tests under argon (Ar) + 5% hydrogen (H_2_) at 1 Bar were conducted at temperatures ranging from 400 to 1050 °C at a deformation rate of 8 × 10^−5^ s^−1^, unless otherwise stated. One tensile test was conducted per set of conditions. An electromechanical tensile machine was used with a 5 kN force sensor. The tensile specimens were cylindrical, with a gage length of 20 mm and a diameter of 3.5 mm, unless otherwise stated. The specimen elongation was considered equal to the crosshead displacement. A bespoke heating system was used to reach and maintain the targeted temperature—this system consists of height elliptical mirrors, each of them containing a 1000 W halogen lamp. The tensile specimen was located at the common focus of the eight mirrors. The temperature was controlled using a type-K thermocouple connected to a feedback loop proportional–integral–derivative (PID) controller. Once the specimen was broken, the furnace was immediately switched off and the specimen was quenched to room temperature within a few minutes, using argon flowing. Scanning electron microscopy was used to observe the fracture surfaces, as well as the sides of the broken specimens. The ductility of the material was evaluated from the reduction in area, measured on the broken tensile specimens in the necking region, if any. The repeatability of this measurement procedure is within ±5% of the reduction in area. Wavelength dispersive spectroscopy (WDS) was used in a few cases to measure the concentration of sulphur segregated on the fracture surfaces of the broken tensile specimens. Multi-voltage measurements were conducted and the data processing method presented in [11] was used, so as to separate the bulk and the surface contributions to the sulphur Kα line measured. The measurements were conducted on large grain boundary areas (50 × 50 µm) in the scanning mode. The presence of an oxide layer covering the fracture surface was evidenced and taken into account in the quantification of sulphur segregation. The tilt angle of the grain boundary facets analysed was taken into account as well. Details about the quantification procedure can be found in [11]. The results are expressed as mass of sulphur per unit surface, which can be converted into a number of sulphur atoms per unit surface and then into a fractional monolayer (it was assumed here that a monolayer has the density of (111) nickel plane).

## 3. Results and Discussion

**Figure 1:**
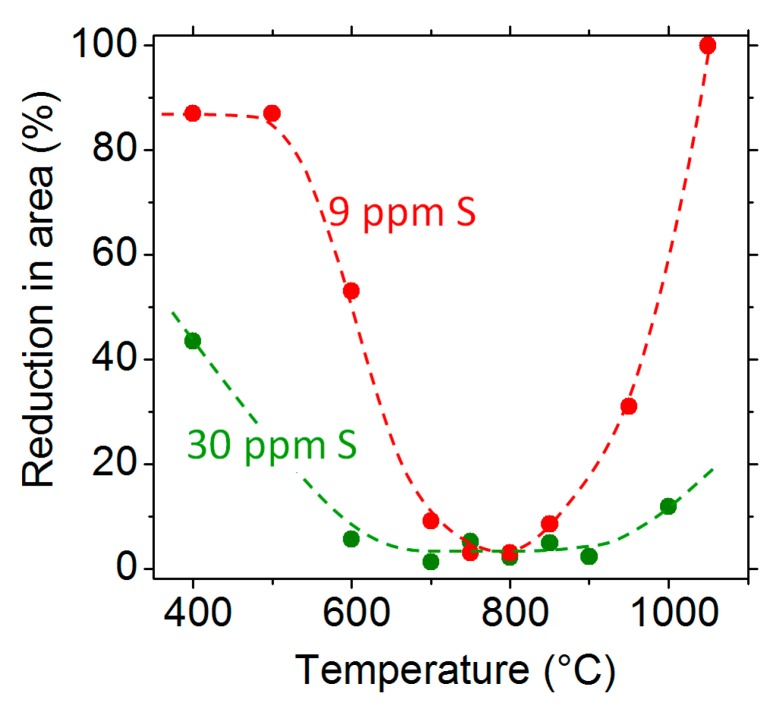
The reduction in area measured after tensile testing at different temperatures for the two sulphur contents.

**Figure 2:**
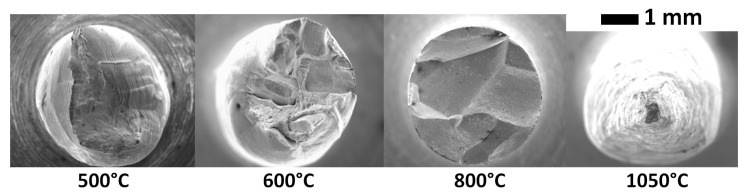
The fracture surfaces of the 9 ppm S specimens, tensile tested at different temperatures.

Figure 1 shows the reduction in area of the tensile specimens tested at different temperatures for the 9 ppm S and the 30 ppm S materials. The effect of the sulphur is clearly evidenced—the ductility trough is wider for the sulphur-enriched material. From 700 to 850 °C, the reduction in area is almost zero for the two materials, which demonstrates their extreme brittleness in that temperature range. The fracture surfaces of the 9 ppm S material at different temperatures are presented in Figure 2. The fracture is ductile at 500 and 1050 °C, which is consistent with the large reduction in area at those temperatures (87% and 100% at 500 and 1050 °C, respectively, see Figure 1). On the other hand, the fracture surface is entirely intergranular at 800 °C, and this is related to the zero reduction in area observed in Figure 1. At 600 °C, the fracture is partly intergranular and partly ductile.

**Figure 3:**
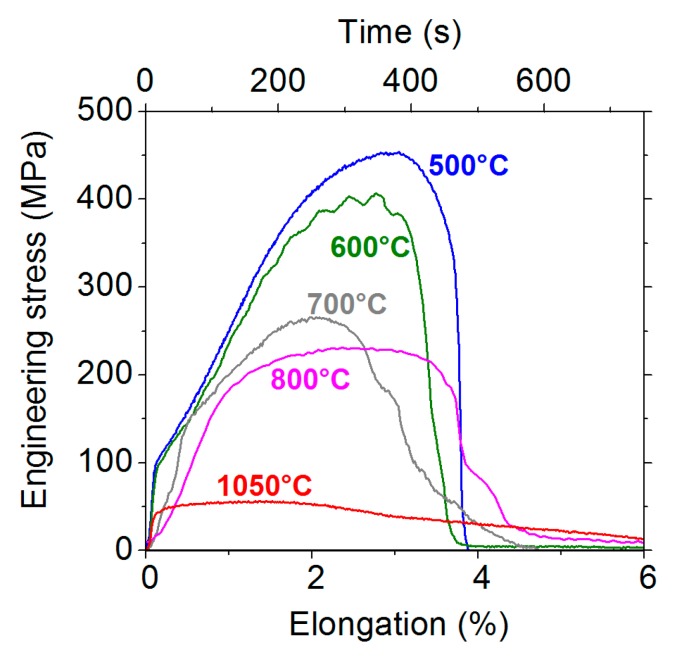
The engineering stress–strain curves of the 9 ppm S material at different temperatures.

Figure 3 shows the engineering stress–strain curves measured on the 9 ppm S materials at different temperatures. As expected, the stress reached during the tensile test decreases when the temperature increases. This is due to the flow behaviour of the material. At 500 and 1050 °C, the decreasing part of the curve is due to the ductile behaviour of the material, which results in strong necking of the specimen (Figure 2). On the other hand, at 700 and 800 °C, no necking is observed (Figure 2). In that case, it is inferred that specimen failure (i.e., the load decrease in Figure 3) is obtained from crack propagation across the specimen. This is consistent with the entirely intergranular fracture surface observed at those temperatures. At 600 °C, the behaviour is intermediate—both necking and intergranular fracture are observed.

**Figure 4:**
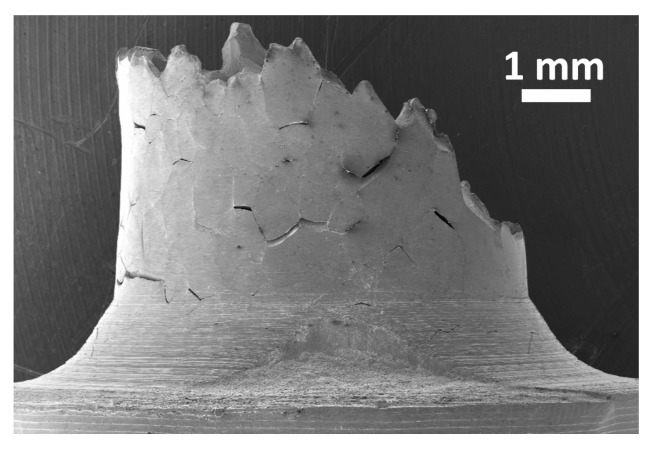
The side view of a 9 ppm S specimen, tensile tested at 750 °C at 8 × 10^−5^ s^−1^. Considerable secondary cracking is observed. A specimen 6 mm in diameter was used here (instead of 3.5 mm) to better evidence the grain boundary nature of the secondary cracks.

Figure 4 shows a side view of the 9 ppm S specimens, tensile tested at 750 °C. Considerable secondary cracking is observed. Systematic observations of the broken specimens show that secondary cracking is always present. This suggests that cracking occurs through a subcritical process, i.e., at a “slow” crack growth rate. In the case of critical cracking (i.e., cracking occurring at the toughness of the material), the crack growth rate is of the order of the speed of sound—in this case, secondary cracking is almost impossible since, once a crack is initiated, the time needed for it to propagate across a millimetric specimen is extremely short (of the order of some microseconds), which allows practically no time to initiate other cracks before complete specimen failure. Thus, the multiple secondary cracking observed here is a clear indication of subcritical, i.e., slow cracking, occurring at a stress intensity factor lower than the toughness of the material. This indicates that the embrittlement is limited to the vicinity of the crack tip. In contrast, embrittlement extended to the whole specimen thickness would result in critical cracking (i.e., at the speed of sound) and no secondary cracking would be observed. In the subcritical regime, the crack growth rate depends not only on the stress intensity factor, but also on the kinetics of crack tip embrittlement. The sulphur here is active at the crack tip and promotes grain boundary failure through a dynamic process. In other words, the grain boundary is embrittled locally, ahead of the crack tip, as the crack propagates. The crack growth rate is limited here by the kinetics of the transport of sulphur to the crack tip. This mechanism can be reasonably described by the “slow fracture” approach proposed by Hirth and Rice [12] and Rice and Wang [13]. In that approach, the grain boundary is fractured, i.e., converted into two surfaces, at constant chemical potential. This implies that the two surfaces created are enriched in the solute (here in sulphur) at the same time as the crack is propagating. This results in a significant reduction in surface energy which, in turn, facilitates crack propagation (in agreement with a simple Griffith criterion, for example, [14]). This process is, of course, possible only if the transport of the solute is fast enough with respect to the crack growth rate. Recently, Kirchheim at al. [15] have proposed the following criterion, which shows that the crack growth rate has to be below a certain threshold to allow such a process:

(1) $$\frac{da}{dt}\leq \frac{2\pi DC_{b}}{\Gamma }$$

where da/dt is the crack growth rate (m s^−1^), D is the solute diffusion coefficient (m^2^ s^−1^), $C_{b}$ is the solute content in solid solution (m^−3^), Γ is the surface solute concentration (m^−2^) covering the surfaces of the crack. The experimental crack growth rate can be roughly estimated from the stress–strain curves presented in Figure 3. For the specimens showing no necking (for example, for the 700 and 800 °C specimens of the 9 ppm S material), the crack length is of the order of the specimen diameter and the propagation time can be estimated as half of the test duration, which is about 500 s. This gives a crack growth rate of the order of 15 µm s^−1^. On the other hand, the threshold crack growth rate (calculated from Equation (1)) is as low as about 2 nm s^−1^ at 800 °C and 2 Å s^−1^ at 700 °C. Those values were obtained using the following inputs: $D=2.6\times 10^{-12}$ cm^2^ s^−1^ at 700 °C and $D=3.2\times 10^{-11}$ cm^2^ s^−1^ at 800 °C (bulk diffusion coefficient of sulphur in nickel [16,17]. The bulk diffusion coefficient of sulphur in gamma iron (instead of nickel) could be considered as well: $D=2.1\times 10^{-12}$ cm^2^ s^−1^ at 700 °C and $D=2.6\times 10^{-11}$ cm^2^ s^−1^ at 800 °C [18]. However, this would not significantly affect the crack growth rate calculated), $C_{b}=9\;\mathrm{wt}\;\mathrm{ppm}=1.4\times 10^{18}$ cm^−3^, $\Gamma =1/3\;\mathrm{monolayer}=6\times 10^{14}$ cm^−2^. The $C_{b}$ term is probably overestimated here, as part of the nominal content is scavenged as manganese sulphides. Considering the actual sulphur content in solid solution would result in even lower crack growth rates. The Γ value was chosen in agreement with the WDS measurements of sulphur concentration on the fracture surfaces presented at the end of this paper. In any case, it is found that the crack is actually propagating faster, by four to five orders of magnitude, than is allowed by the criterion (shown in Equation 1). One possible explanation for this is that the transport of the solute is actually strongly accelerated by plastic deformation. This would be consistent with the huge acceleration of sulphur diffusion in nickel during plastic deformation observed by Allart et al. [19]. Those authors showed that the transport of sulphur in nickel in the 450–550 °C temperature range was strongly correlated to plastic deformation and was faster, by four to five orders of magnitude, than normal bulk diffusion. Although the temperatures investigated here are slightly higher than in the study of Allart et al., it is likely that the same acceleration phenomenon occurs here, due to plastic deformation. This acceleration of the solute transport allows a fast crack growth rate (according to Equation (1)).

**Figure 5:**
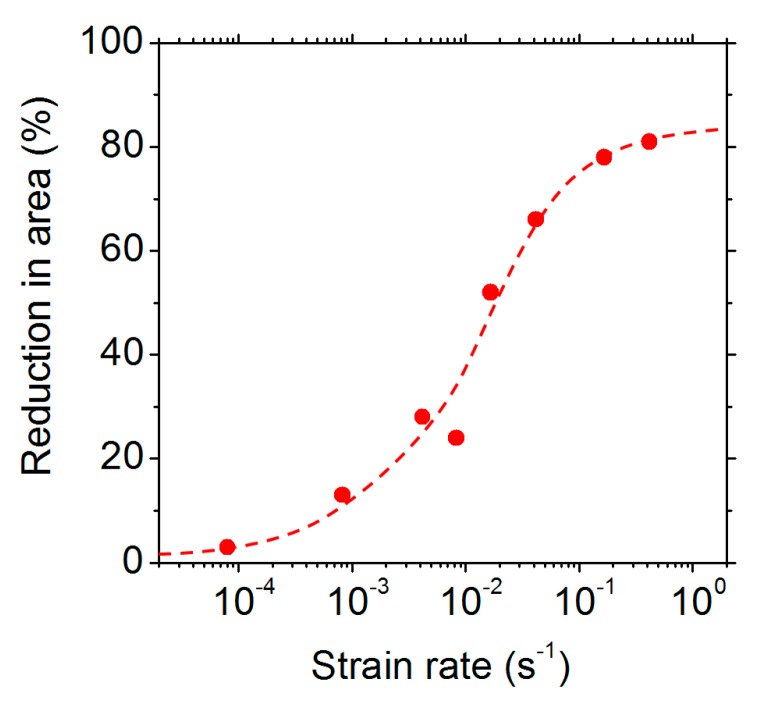
The reduction in area observed after tensile testing at 850 °C of the 9 ppm S material at different strain rates. A pre-heating step at 850 °C was conducted so that the total time spent at 850 °C for all the specimens was 40 min.

In order to investigate the effect of the strain rate on the ductility trough, tensile tests were conducted at 850 °C on the 9 ppm S material at different strain rates. A pre-heating step at 850 °C was used so that the total time spent at that temperature (pre-heating time + tensile test time) was the same for all the specimens, whatever the strain rate used. This total time was 40 minutes. Figure 5 shows the reduction in area obtained as a function of the strain rate. A very strong effect of the strain rate is observed—the material is completely brittle at a low strain rate, but the ductility is recovered at a high strain rate.

**Figure 6:**
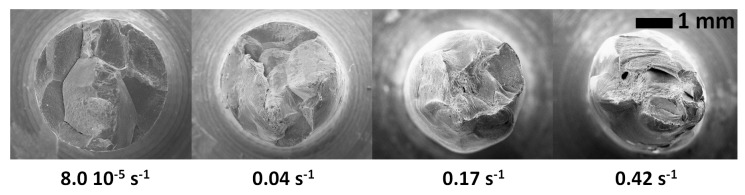
The fracture surfaces of the 9 ppm S specimens, tensile tested at 850 °C at different strain rates.

**Figure 7:**
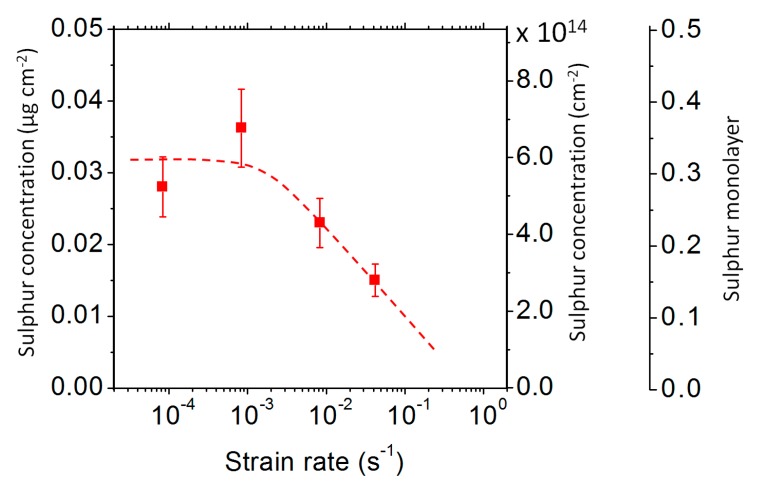
The sulphur concentration measured using wavelength-dispersive spectroscopy (WDS) on the fracture surface of the 9 ppm S specimens, tensile tested at 850 °C at different strain rates.

Figure 6 shows the fracture surfaces obtained at different strain rates. The fracture is ductile at the two highest strain rates (0.17 and 0.42 s^−1^) and entirely intergranular at the lowest one (8 × 10^−5^ s^−1^). It is remarkable that, at a strain rate as high as 0.04 s^−1^, the fracture is still significantly intergranular. The amount of sulphur segregated on the fracture surfaces was measured using post-mortem WDS analysis for three different strain rates. The results are shown in Figure 7. For the lowest strain rates, the sulphur concentration is about 0.03 µg cm^−2^, which corresponds to a 0.3 monolayer. For higher strain rates, the sulphur concentration decreases. However, it must be noted that the sulphur concentration on the fracture surface of the specimen that was tensile tested at 0.04 s^−1^ is as high as 0.15 monolayer, while the duration of the tensile test was only a few seconds. This, again, shows that the transport of sulphur was extremely fast.

A possible interpretation of those results is based on the sulphur transport by moving dislocations. At low and intermediate strain rates (from 8 × 10^−5^ to 0.04 s^−1^), dislocations move slowly enough to carry some sulphur to the propagating crack tip. This is why sulphur can be detected on the fracture surface. It is remarkable that, at 850 °C, this dragging mechanism is still significant at strain rates as high as 0.04 s^−1^. At the highest strain rates, however (0.17 and 0.42 s^−1^), this mechanism is not possible any longer, because the dislocation velocity becomes too high to drag the solute. In that case, the intergranular fracture is not possible anymore and the ductility is recovered.

Finally, it must be noted that the embrittlement observed here is independent of the time spent at a given temperature (as stated, the total time spent at 850 °C was the same for all the tests in Figure 5). This shows that the embrittlement is not related to any possible static grain boundary segregation that existed before mechanical loading.

## 4. Conclusions

The ductility trough was studied for two iron–nickel alloys with different sulphur contents. The main conclusions of this work can be summarised as follows:

1. Sulphur has a strong detrimental effect on the hot ductility of the alloys;
2. The loss of ductility is due to a dynamic embrittlement caused by sulphur at the crack tip, resulting in subcritical crack growth;
3. Highly accelerated transport of sulphur to the crack tip due to plastic deformation allows high crack growth rates, even at “low” temperatures and/or at “high” strain rates. It is suggested that this acceleration is due to the dragging of sulphur by moving dislocations;
4. The sulphur concentration measured on the fracture surface decreases with the increasing strain rate;
5. Ductility is recovered at high strain rates, which is consistent with the sulphur dislocation dragging mechanism.

## Figures and Tables

**Table 1:** The composition of the two materials used in this study. All the data are given in wt.%. Iron (Fe), nickel (Ni), manganese (Mn) and silicon (Si) contents were obtained from X-ray fluorescence and sulphur (S) and carbon (C) contents from glow discharge mass spectrometry (GDMS).

Material	Fe	Ni	Mn	Si	S	C
9 ppm S	Bal.	41.95	0.412	0.117	0.0009	0.003
30 ppm S	Bal.	41.93	0.413	0.104	0.0030	0.005
